# Albuminuria, Hypertension, and Reduced Kidney Volumes in Adolescents Born Extremely Premature

**DOI:** 10.3389/fped.2020.00230

**Published:** 2020-05-12

**Authors:** Keia R. Sanderson, Emily Chang, Erica Bjornstad, Susan L. Hogan, Yichun Hu, David Askenazi, Rebecca C. Fry, T. Michael O'Shea

**Affiliations:** ^1^Department of Medicine-Nephrology, University of North Carolina, Chapel Hill, NC, United States; ^2^Division of Pediatric Nephrology, University of Alabama at Birmingham, Birmingham, AL, United States; ^3^Department of Environmental Sciences and Engineering, Gillings School of Global Public Health, University of North Carolina, Chapel Hill, NC, United States; ^4^Division of Neonatal-Perinatal Medicine, Department of Pediatrics, University of North Carolina, Chapel Hill, NC, United States

**Keywords:** chronic kidney disease, pediatric, proteinuria, hypertension, kidney volume reduction, kidney ultrasound, preterm birth, extremely preterm birth

## Abstract

**Background:** Premature birth is associated with decreased nephron number and an increased risk for chronic kidney disease (CKD). To inform the development of guidelines for kidney follow up of children born prematurely, we undertook a study of individuals born extremely preterm, with the aim of characterizing the prevalence and predictors of microalbuminuria, elevated blood pressure, and/or abnormal kidney volume in adolescence.

**Methods:** Study participants (*n* = 42) were born before 28 weeks of gestation and were enrolled at birth in the Extremely Low Gestational Age Newborns (ELGAN) study. When participants were 15 years old, we obtained 2 manual blood pressures, a spot urine microalbumin measurement, and sonographic measurements of kidney length and volume.

**Results:** Of the 42 participants, 60% were male, 52% were Caucasian (18% Hispanic), and 43% were African-American. Their median age was 15 (IQR 15, 15.3) years. In 33.3% of the cohort, blood pressure was elevated (>120/80 mmHg). Microalbuminuria (>30 mg/g) was present in 11.9% of the cohort, and kidney volume below the 10th percentile of normative data was present in 14%. Twenty-one (50%) of the sample had at least one kidney abnormality (microalbuminuria, elevated blood pressures, and/or kidney hypoplasia); these individuals were more likely to have experienced neonatal hypotension [55% vs. 17% among those with no kidney abnormality, *p* = 0.02].

**Conclusions:** Half of adolescents in this subset of ELGAN cohort have at least one risk factor of kidney disease (reduced kidney volume, microalbuminuria, and/or elevated blood pressures) at 15 years of age. This study suggests the importance of monitoring kidney outcomes in children after extremely preterm birth, especially those with a history of neonatal hypotension.

## Introduction

Pediatric chronic kidney disease (CKD) carries a mortality rate 1000-fold higher compared to the age-matched non-CKD population, yet the initial onset of CKD is asymptomatic, delaying diagnosis in pediatric patients (1). Delay in early recognition of CKD is associated with more rapid disease progression and earlier mortality among patients with End Stage Kidney Disease (ESKD) (2, 3).

Preterm birth, which accounts for 10% of all births in the US, is associated with a near doubling of the risk of CKD (4–7). Harer et al. demonstrated that nearly a quarter of very low birth weight infants had evidence of kidney dysfunction during early childhood and South et al. found that adolescents with a history of prematurity had higher blood pressures and decreased kidney function compared to adolescents born at term (8, 9). Incomplete nephrogenesis and the resultant poor nephron endowment lead to proteinuria, hypertension, nephrotoxic inflammation, tissue remodeling, and renal tubulo-interstitial fibrosis (8–12). However, not all persons born with a reduced number of nephrons (e.g., solitary kidney) develop CKD suggesting that additional risk factors during the critical period of postnatal nephron maturation and growth could contribute to progressive loss of kidney function (10, 13–15).

Despite the increased risk of CKD among individuals born preterm, evidenced-based guidelines have not been established for kidney follow up of preterm infants after discharge from the neonatal intensive care unit (NICU). Such guidelines might allow for earlier diagnosis and application of therapies which slow progression to ESRD (16, 17).

To inform the development of guidelines for kidney follow up of individuals born extremely preterm, we evaluated kidney outcomes in a sample of 15-year-old adolescents born extremely preterm (prior to the third trimester) (18). Our objectives were to evaluate, at 15 years of age, the prevalence of markers of early evidence of kidney disease (microalbuminuria, hypertension, and reduced kidney volume) and the association between perinatal predictors.

## Methods

This is a prospective cohort study approved by the Institutional Review Board of the University of North Carolina at Chapel Hill (UNC-CH).

### ELGAN Study Participants

Study participants were a subset of individuals (*n* = 42) enrolled in the Extremely Low Gestational Age Newborn (ELGAN) study. The ELGAN study is a multicenter prospective observational study of infants born before 28 weeks gestation between April 2002 and August 2004. Infants were enrolled at birth at 14 hospitals in 5 states. The details of the population, IRB approvals, consent, and methods have been previously described (18).

For the current study, we consecutively enrolled 42 ELGAN study participants around the time of their 15th birthday at a single site (University of North Carolina-Chapel Hill). Study visits were completed between September 1, 2017 and April 31, 2019. A total of 53 ELGAN study participants were evaluated at 10 years of age at the University of North Carolina-Chapel Hill and were eligible for the current study; 42 (79%) were enrolled. Eleven potential participants were not enrolled due to lack of transportation to the study site and/or scheduling conflicts. No participants met criteria for exclusion, which included active pregnancy or fever.

**Figure 1:**
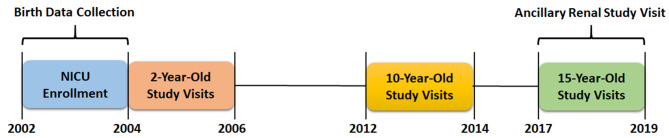
ELGAN study data collection timeline.

Figure 1 depicts the time course for data collection from the original ELGAN study and for the ancillary kidney study visit.

### Birth Characteristics From the ELGAN Study, 2002–2004

Selected characteristics of the newborn period were abstracted from the ELGAN database originally collected during each participant's neonatal intensive care unit (NICU) hospital course from 2002 to 2004. The neonatal characteristics evaluated in the current study were selected based upon prior studies indicating a relationship between these neonatal characteristics and nephrotoxicity or neonatal acute kidney injury (AKI) and CKD (15, 19, 20). The following were abstracted from the ELGAN study database: (1) delivery resuscitation interventions, (2) APGAR scores, (3) diagnosis of sepsis within the first 28 days of life, (4) number of mechanical ventilator days in the first 28 days of life, (5) receipt of indomethacin for patent ductus arteriosus (PDA) at any time during their NICU course, (6) urine output in the first 12 h of life was recorded (converted to ml/kg/hr for analysis), (7) having an elevation (defined below) of a systemic inflammatory marker (CRP, IGF, EPO, VEGFR, IL-8, TNF-α, IL-6, and IL-1β), lowest mean arterial pressure(MAP) in the first 28 days of life, (9) diagnosis of neonatal hypotension as defined by a lowest MAP in lowest quartile for gestational age, (10) use of vasopressors in the first 14 days of life, (11) prolonged patent ductus arteriosus, (12) number of doses of methylxanthines in the first 28 days of life, (13) growth velocity throughout NICU hospital course (grams/kg/day), (14) length of NICU course, and (15) gestational age at birth and at hospital discharge (18).

Details of measurements of inflammatory proteins are previously published (21). An elevation of an inflammatory protein was defined as a concentration in the top quartile for gestational age and postnatal day, and participants with one or more inflammatory protein elevations within the first two postnatal weeks were classified as having neonatal systemic inflammation.

### Kidney Measurements Collected During ELGAN 15 Year-Old Kidney Study Visit, 2017–2019

The primary outcome was the presence of the composite kidney outcome as defined by one or more of the following: microalbuminuria, hypertension, and or reduced kidney volume, based on evidence that individuals with these biomarkers are at an increased risk for progressive CKD (estimated GFR <90 ml/min/1.73 m2) during childhood (17, 22–24).

Urine albumin/creatinine was measured in a random 1 mL urine clean catch sample which was refrigerated at 0–4.5 degrees Celsius and delivered to UNC-CH McLendon lab to be processed within 12 h of sample collection. Urine creatinine was measured enzymatically using the VITROS creatinine microslide (Mfr. No. 6802584) which uses an IDMS traceable method with calibration to the National Institute of Standards and Technology (NIST) SRM® 914 creatinine reference standard. Urine albumin was measured turbidometrically using antibodies specific for human albumin (Mfr. No. 6801740). Immunochemical reactions of the antibody/antigen complexes increase solution turbidity which is measured spectrophotometrically at 340 nm. Testing for both urine creatinine and urine albumin were performed in the UNC-CH McLendon Laboratory on the VITROS 5600 System. Microalbuminuria was defined as >30 μg albumin/milligram creatinine (25). Two manual blood pressures were measured after 5 min of rest on the right upper extremity using FDA approved MDF Instruments aneroid sphygmomanometer with appropriately sized cuffs. Two blood pressures were measured 5 min apart while the study participants were seated with feet flat on the floor and were averaged for the final blood pressure measurement. Participants were classified as having hypertension if the mean of two blood pressure measurements were greater than the 95th percentile for age, sex, and height percentile or >120/80 mmHg consistent with the 2017 American Academy of Pediatrics Clinical Practice Guidelines for Screening and Management of High Blood Pressure in Children and Adolescents (26).

Kidney volume was measured by ultrasonography using the Sequoia S512 System with a 4C1 transducer and physician ultrasonographers certified by Emory University School of Medicine, “Ultrasonography for Nephrologists” course (27). Each participant underwent renal ultrasound measurement of renal length, width, and anterior-posterior diameter of both right and left kidneys. All kidney dimensions were the maximum values of the kidney with the patient lying in a prone position.

Kidney volume was calculated according to the volume formula of a rotational ellipsoid:

RV[mL]=(Length*Width*Depth(anterior-posterior diameter)) *π^**6**^ (28, 29).

Body surface area (BSA) was calculated according to D. Du Bois and E.D. Du Bois as:

**BSA[m2]=(body weight[kg]**^0.425^*body height[cm]^**0.725**^**)** *0.007184 (30).

BSA related renal volume (BSARV) was calculated as:

**BSARV (ml/m**^**2**^**)** **=** **RV(mL)/BSA(m**^**2**^**)**.

Total kidney volume per body surface area was calculated by summation of right and left kidney volumes then individualized to body surface area:

**(TKV/BSA** **=** **ml/m2)**.

Decreased kidney volumes or kidney hypoplasia was defined by participants having TKV/BSA below the 10th percentile of normative TKV/BSA per previously published data which suggests that individuals with TKV/BSA in this range have an inherent risk for oligonephropathy (28, 29, 31–33).

### Statistical Analysis

Descriptive statistics included frequencies with percentage of categorical variables and mean with standard deviation (SD) or median with interquartile rang (IQR) of measures of central tendency for continuous variables. Participants were then categorized as having or not having the composite renal outcome, as defined above. To evaluate directions of associations and potential impacts of nephrotoxic neonatal exposures in this small sample we used chi-square and Fisher's exact test of significance for categorical exposures. For continuous exposures, we used Mann–Whitney *U*-test (for independent variables with non-normal distribution) or *t*-tests (for independent variables that were normally distributed).

The comparisons were done between renal volume in the ELGAN sample and previously published normative data on renal volume (28, 29, 31). *P*-values were calculated by *t*-test for normal distribution.

Univariate logistic regression models were used to estimates of the strength of association between neonatal exposures and the composite renal outcome. Given the size of this sample, we did not perform multivariable analyses. Statistical significance was defined as a *p*-value of < 0.05, but our main goal was to evaluate direction and potential impacts for guiding future research, including calculation of sample sizes needed. All analyses were conducted with SAS (v.9.4, Cary, N.C.).

## Results

### Study Participants

The median age of the cohort was 15.0 (IQR 15.0, 15.3) years, 59.5% were male, 52.38% were Caucasian (18.1% Hispanic), and 42.9% were African-Americans. The mean body mass index (BMI) percentile was at the 71st percentile for age and sex and 52.4% participants were overweight (BMI greater than the 85th percentile for age and sex) (34). Mean gestational age a birth was 25.7 (±1.1) weeks and mean birth weight was 770 (±173) grams (Table 1).

**Table 1:** ELGAN study birth characteristics collected in 2002–2004 and ancillary kidney study visit data collected in 2017–2019 by composite kidney outcome (microalbuminuria, elevated blood pressure, and/or abnormal kidney mass by ultrasound^*^).

**Characteristics**	**Total** **(*n* = 42)**	**Composite kidney outcome**	**Composite kidney outcome**	*P*-value^***^
		**Absent** **(*n* = 21)**	**Present** **(*n* = 21)**	
**Birth characteristics collected 2002–2004**				
Gestational age at birth (weeks), mean(SD)	25.7 ± 1.1	25.5 ± 1.0	26.0 ± 1.1	0.13
Birth weight (grams), mean(SD)	770.0 ± 173.1	807.2 ± 190.3	732.8 ± 149.3	0.22
Delivery resuscitation interventions, *n* (%)				0.94
Missing	1 (2.4%)	0 (0.0%)	1 (4.8%)	
Positive pressure ventilation	5 (11.9%)	3 (14.3%)	2 (9.5%)	
Intubation	33 (78.6%)	16 (76.2%)	17 (81.0%)	
Compressions	1 (2.4%)	1 (4.8%)	0 (0.00%)	
Epinephrine	2 (4.8%)	1 (4.8%)	1 (4.8%)	
APGAR 1 min, md(IQR)	4.5 (2.0, 7.0)	5.00 (2.0, 6.0)	4.0 (3.0, 7.0)	0.45
APGAR 5 min, md(IQR)	7.0 (5.0, 8.0)	7.0 (5.0, 8.0)	7.00 (6.00, 8.00)	0.56
Neonatal indomethacin exposure, *n* (%)	19 (45.2%)	11 (52.4%)	8 (38.1%)	0.35
Neonatal sepsis in 1st month, *n* (%)				0.70
Missing	1 (2.4%)	1 (4.8%)	0 (0.0%)	
No	31 (73.8%)	16 (76.2%)	15 (71.4%)	
Yes	10 (23.8%)	4 (19.1%)	6 (28.57%)	
Mechanical ventilator days, md (IQR)	19.0 (5.0, 28.0)	26.5 (8.0, 28.0)	14.0 (4.0, 28.0)	0.31
Urine output (ml/kg/hr), mean(SD) *^δ^*	1.6 ±1.1	1.7 ± 1.3	1.4 ± 0.9	0.11
Any inflammatory proteins >75th percentile, *n* (%)^¥^	26 (61.9%)	12 (57.1%)	14 (66.7%)	0.75
Duration of NICU course (weeks), mean(SD)	12.7 ± 6.9	14.2 ± 8.3	11.1 ± 4.8	0.06
Lowest MAP(mmHg), med (IQR)	23.0 (19.0, 27.0)	23.0 (21.0, 29.0)	20.0 (17.0, 25.0)	0.10
Hypotension, *n* (%)	16 (38.1%)	5 (23.8%)	11 (52.4%)	0.0236
Use of vasopressors in first 14 days of life, *n* (%)	21 (50%)	8 (38.1%)	13 (61.9%)	0.35
Any patent ductus arteriosus, *n* (%)	28 (66.7%)	12 (57.1%)	16 (76.2%)	0.21
^#^ of Doses of methylxanthine in first 28 days, md(IQR)	10.0 (0.0, 24.0)	5 (0.0, 22.0)	12.0 (2.0, 25.0)	0.11
Growth velocity (g/kg/day), mean(SD)^**^	24.6 ± 8.6	24.7 ± 8.7	24.6 ± 8.7	0.64
Gestational age at discharge, mean(SD)	38.4 ± 6.5	39.7 ± 7.9	37.1 ± 4.3	0.09
**Ancillary 15-year old renal study visit characteristics collected 2017–2019**				
Age (years), med (IQR)	15.0 (15.0, 15.3)	15.0 (15.0, 15.1)	15.1 (15.0, 15.4)	0.47
Males, *n* (%)	25 (59.5%)	10 (47.6%)	15 (71.4%)	0.35
Race, *n* (%)				0.67
Caucasian	22 (52.4%)	11 (52.4%)	11 (52.4%)	
African-American	18 (42.9%)	9 (42.9%)	9 (42.9%)	
Other	2 (4.8%)	1 (4.8%)	1 (4.8%)	
BMI >85th percentile, *n* (%)	22 (52.4%)	10 (47.6%)	12 (57.1%)	0.53
BMI percentile, mean(SD)	70.7 ± 30.2	70.2 ± 29.8	71.1 ± 31.0	0.92
Elevated blood pressure (>120/80 mmHg), *n* (%)	14 (33.3%)	0 (0.0%)	14 (58.3%)	–
Systolic blood pressure (mmHg), med (IQR)	115.8 (109.0, 121.5)	111.0 (107.0, 116.0)	121.0 (111.0, 125.5)	0.0021
Diastolic blood pressure (mmHg), med (IQR)	70.0 (64.0, 76.0)	69.5 (64.0, 73.0)	71.0 (66.0, 81.0)	0.25
Renal hypoplasia^*^, *n* (%)	6 (14.3%)	0 (0.0%)	6 (28.6%)	–
Right renal length (cm), mean(SD)	9.4 ± 0.7	9.4 ± 0.9	9.3 ± 0.6	0.12
Left renal length (cm), mean(SD)	9.7 ± 0.9	9.8 ± 1.0	9.6 ± 0.8	0.09
Microalbuminuria (>30μg/g), *n* (%)	5 (11.9%)	0 (0.0%)	5 (23.8%)	–
Urine albumin/creatinine (μg/g), md(IQR)	7.6 (5.4, 14.9)	6.7 (4.6, 11.8)	12.5 (5.9, 30.7)	0.0253

* Abnormal renal mass defined by body surface area related renal volume below mean and standard deviation.

δ Urine output in the first 12 h of life.

¥ Inflammatory proteins measured at birth include CRP, IGF, EPO, VEGFR, IL-8, TNF-α, IL-6, and IL-1β.

** Growth Velocity = [(Discharge weight-birthweight)/birthweight]/Length of Stay (18).

# lowest MAP in lowest quartile for gestational age.

*** *P-values were calculated by Chi-square and Fisher's exact test of significance for categorical independent variables, Mannz–Whitney U-test for non-parametric interval independent variables, t-test of significance used for interval independent variables*.

### Kidney Outcomes

The median systolic and diastolic blood pressures of the ELGAN cohort were 115.8 (IQR: 10,9122) and 70 (IQR: 6,476) mmHg. While the overall mean systolic and diastolic blood pressures were within the normal range, 33.3% of the cohort had elevated systolic and/or diastolic blood pressures (>120/80 mmHg). The median systolic blood pressure for adolescents with the presence of the composite kidney outcome was significantly elevated [121 (IQR: 111,125.5) mmHg] compared to a mean systolic blood pressure of 111 (IQR: 107,116) mmHg among adolescents without the composite kidney outcome (*p* < 0.01). However, no difference was found when comparing diastolic blood pressure for those with and without the composite kidney outcome. Of the adolescents with systolic and/or diastolic blood pressure >120/80 mmHg, the mean systolic blood pressure was 124.68 ± 3.4 mmHg and 76.18 ± 9.6 mmHg.

Median spot urine albumin/creatinine ratio was 7.6 (IQR: 5.4, 14.9) mg/g. However, 11.9% of the cohort demonstrated evidence of microalbuminuria (>30 mg/g). Among the nine participants with only microalbuminuria, the mean urine albumin/creatinine was 95.4 ± 72.8 mg/g.

When comparing this ELGAN study cohort to age and sex-matched normative data (28, 29, 31), there were no clinically or statistically significant differences in kidney length and volume (Table 2). Mean right and left kidney length were 9.4 (±0.7) cm and 9.7 (±0.9) cm, compared to normative kidney length data (normative right kidney 9.2 ± 0.7 cm (*p* = 0.54), normative left kidney 9.9 ± 0.6 cm) (*p* = 0.46), respectively. Mean total kidney volume per body surface area (TKV/BSA) was 126.4 (±26.7) ml/m^2^ compared to the pediatric normative mean TKV/BSA (132 (±31.3) ml/m^2^) (*p* = 0.19).

**Table 2:** Mean (SD) renal ultrasound measurements from adolescent participants of the UNC-CH ELGAN study who participated in a kidney study visit (2017–2019), compared to mean (SD) normative age-matched kidney ultrasound data^*^.

	**Composite kidney outcome**	**Composite kidney outcome**	**ELGAN kidney study**	Normative data^*^	*P*-value^***^
	**Absent** ***n* = 21**	**Present** ***n* = 21**	***n* = 42**	n^**^	
Right kidney length (cm)	9.4 ± 0.9	9.3 ± 0.6	9.4 ± 0.7	9.2 ± 0.7	0.54
Right body surface area related kidney volume (ml/m^2^)	61.6 ± 10.0	62.1 ± 20.3	61.8 ± 15.8	65.4 ± 15.2	0.14
Left kidney length (cm)	9.8 ± 1.0	9.6 ± 0.8	9.7 ± 0.9	9.9 ± 0.6	0.46
Left body surface area related kidney volume (ml/m^2^)	66.0 ± 14.0	63.1 ± 15.1	64.6 ± 14.5	66.3 ± 15.9	0.49
Total kidney volume per body surface area (ml/m^2^)	127.6 ± 19.7	125.2 ± 32.7	126.4 ± 26.7	132 ± 31.3	0.26

* Normative data references (28–30).

** n, The normative data sample size for kidney lengths is 11 and the normative data sample size for kidney volumes is 624.

*** *P-values were calculated, by t-test for comparison between ELGAN Cohort and Normative data*.

Overall, 50% of the adolescent cohort (*n* = 21) had one or more biomarkers of CKD as evidenced by elevated blood pressures, microalbuminuria, and or reduced kidney volume measurement by kidney ultrasound. None of the participants had a prior knowledge of a history of kidney disease, elevated blood pressures, proteinuria, or known structural abnormalities of the kidney, and/or urinary tract. Kidney volumes among the adolescents with and without the presence of the composite kidney outcome (microalbuminuria, hypertension, and or reduced kidney volumes) were not statistically different [TKV/BSA 125.2 ± 32.7 vs. 127.6 ± 19.7 ml/m^2^ vs., respectively (*p* = 0.09)]. However, 14.3% of the cohort demonstrated kidney volume less than the 10th percentile BSA normalized kidney volume normative data, a marker previously reported to be associated with oligonephropathy (Table 2) (32). Of note, there were no statistical differences in the presence of albuminuria, hypertension, and/or reduced kidney volume by race, overweight status, or sex (Table 1). Of note, we did not find differences in the presence of albuminuria, hypertension, and/or reduced kidney volume by race, overweight status, or sex.

### Neonatal Risk Factors

We compared birth and demographic characteristics in the adolescents with and without the composite kidney outcome of elevated blood pressure, microalbuminuria, and/or abnormal kidney volume (Table 1). Adolescents with the composite outcome, as compared to those without this outcome had similar gestational ages at birth (26.0 ± 1.1 vs. 25.4 ± 1.1 weeks, *p* = 0.13) and birth weights (741.4 ± 142.4 vs. 808.1 ± 205.3 grams, *p* = 0.22) (Table 1). However, the frequency of neonatal hypotension (as defined by having a lowest MAP in the lowest quartile for age) was significantly greater (55 vs. 17%, *p* = 0.02) in those with the presence of the composite kidney outcome. We found no other neonatal clinical differences between adolescents with and without the composite kidney outcome.

In the univariate logistic regression analyses, there were no neonatal characteristics that were statistically significantly associated with the composite kidney outcome although neonatal hypotension approached significance with a wide confidence interval (OR 3.52, 95%CI 0.94, 13.17; *p* = 0.06).

## Discussion

The most important finding from this study is that in a sample of adolescents born extremely preterm, 50% had one or more abnormalities associated with CKD (microalbuminuria, elevated blood pressures, and/or reduced kidney volumes). This prevalence is higher than the sum of the prevalence's of microalbuminuria (<3%), hypertension (3–5%), and congenital kidney anomalies (<1%) reported in the general pediatric population (26, 35–37).

As compared to prior studies involving children born preterm, our study demonstrates a higher prevalence of elevated blood pressures and microalbuminuria. In a sample of 422 young adults born before 32 weeks of gestation, 10% had elevated blood pressure and 2.7% had microalbuminuria, as compared to 33.3% with elevated blood pressures and 11.9% with microalbuminuria in the sample described here (38, 39). Likely explanations are that the mean gestational age and birth weight were lower in our sample as compared to those studied previously, and the relatively high frequency of African-American (42.9%) and overweight (53.8%) children in our cohort (34, 40–46). Both overweight status and African-American race are independent risk factors for CKD related to hyperfiltration, obesity related focal segmental glomerulosclerosis, and the genetic predisposition to develop CKD (6, 44, 47).

In contrast to prior studies, we found no difference between total kidney volume per BSA(TKV/BSA) in children born extremely premature, as compared to normative pediatric kidney ultrasound data (22, 48–51). Nearly 60% of human nephrons are formed during the second and third trimesters of gestation, and renal autopsy studies have shown that the total number of nephrons correlates with gestational age at birth (10–12). Infants born prematurely, and thus during a period of relatively rapid nephrogenesis have reduced nephron endowment, reduced kidney volume, and increased risk for CKD long-term (5, 11, 52). On the other hand, some infants born prematurely achieve normal TKV/BSA post-term (33). Our finding that children with TKV/BSA below the 10th percentile of normative data had a greater decrease in kidney size in the left kidney length compared to right kidney length is consistent with findings from a study of kidney length and volumes among young adults born preterm (22).

We found that none of the hypothesized neonatal risk factors were associated with the presence of one or more abnormal kidney outcome (microalbuminuria, elevated blood pressures, and/or reduced kidney volume) during adolescence. Yamamura-Miyazaki et al. found that a lower 5-min Apgar was associated with decreased cystatin C-estimated glomerular filtration rate in school aged children after preterm birth (46). This could not be assessed here as glomerular filtration rate was not assessed in the ELGAN sample. A history of neonatal hypotension was more prevalent among adolescents in this cohort with the composite kidney outcome, but an association between neonatal hypotension and an abnormal kidney outcome was not significant in the univariate analyses. Further study of these risk factors is needed in large samples, as hypotension after preterm birth might compromise renal perfusion during a critical period of post-natal nephron development and maturation. Studies with larger sample sizes could identify risk factors for adverse kidney outcomes so that infants with these risk factors can be more frequently monitored after discharge from the neonatal intensive care unit.

Strengths of our study include selection of a cohort based on gestational age, not birth weight, which minimized confounding due to factors related to growth restriction (53). Another strength is the use of non-invasive markers of kidney disease that have been shown to be predictive of long term CKD and more rapid progression of kidney disease (23, 24). With advancements in ultrasonography in outpatient office settings, the approach we used to evaluate kidney outcomes could serve as a non-invasive screening approach for use by primary care physicians and nephrologists.

The study has limitations including the small sample size, which reduces the generalizability of the study and the statistical power to detect associations with early life exposures. Serum creatinine and cystatin-c are more widely accepted and valid markers for evaluation of kidney dysfunction, but we did not collect blood samples from study participants. Spot urine microalbumin to creatinine samples were used to support rapid screening of patients in this study, however, this could potentially overestimate the frequency of microalbuminuria due to potential contribution of orthostatic proteinuria and other causes of albuminuria. Elevated blood pressures were diagnosed by manual blood pressure measurements though 24-h ambulatory blood pressure monitoring (24 hr ABPM) studies are the preferred method for diagnosis of systemic hypertension. In future studies, first morning urine samples and 24 hr ABPM studies should be obtained to reduce potential misclassifications.

In conclusion, our study suggests that children born extremely prematurely are at increased risk for developing elevated blood pressures, microalbuminuria, or decreased kidney volumes during adolescence. We hope this preliminary study will lead to future studies with larger samples sizes to determine whether our findings can be replicated in other samples of individuals born extremely preterm and to better define neonatal exposures which might compound the risk for kidney disease during childhood. If the larger studies that we intend to pursue replicate our preliminary study findings of increased risk for CKD among children born extremely preterm, this would provide greater evidence to support early and perhaps annual screening for these children such that early interventions, which delay the progression of CKD, can be effectively employed.

Finally, although we and others continue to document that extremely low gestational age newborns are at risk for CKD, to date there are no evidence-based consensus guidelines for follow up of kidney health after extremely preterm birth. Larger, long-term multi-site studies of individuals born extremely preterm will help determine the best evidence-based approach to kidney follow up after extremely preterm birth. Currently, the AAP recommends after discharge from neonatal intensive care, premature infants should have monitoring of blood pressure and growth with every well-child care visit (26). We believe that this recommendation should also be followed for each visit to high risk infant follow-up clinics. We believe that additional monitoring of serum creatinine, electrolytes, kidney ultrasound, and urine microalbumin should be performed in premature infants at well-child care visits, especially if they have a history of acute kidney injury, are extremely premature, small for gestational age, intrauterine growth restricted, and/or if they have other risk factors for CKD (e.g., congenital kidney abnormalities, diabetes mellitus), chronic medical comorbidities(e.g., chronic lung disease, congenital heart disease), overweight/obesity (BMI >85th percentile), and/or BP >95th percentile. We emphasize that these approaches are not consensus guidelines but rather, are based on opinions of authors. There is a great need to further study which premature infants, which tests, and what recommendations should be provided to those at risk for CKD and those with CKD for individuals born extremely preterm.

## Data Availability Statement

The datasets generated for this study are available on request to the corresponding author with consideration of privacy and ethical restrictions.

## Ethics Statement

The studies involving human participants were reviewed and approved by University Of North Carolina- Chapel Hill. Written informed consent to participate in this study was provided by the participants' legal guardian/next of kin.

## Author Contributions

KS, TO'S, EB, and EC contributed conception and design of the study. KS organized the database and wrote the first draft of the manuscript. SH and YH performed the statistical analysis. KS, TO'S, DA, EC, EB, YH, SH, and RF wrote sections of the manuscript. All authors contributed to manuscript revision, read, and approved the submitted version.

## Conflict of Interest

The authors declare that the research was conducted in the absence of any commercial or financial relationships that could be construed as a potential conflict of interest.
